# DPP Inhibition Enhances the Efficacy of PD-1 Blockade by Remodeling the Tumor Microenvironment in Lewis Lung Carcinoma Model

**DOI:** 10.3390/biom14040391

**Published:** 2024-03-25

**Authors:** Mengrong Lei, Junyan Liu, Ying Gao, Wenting Dai, Hanxue Huang, Qingqing Jiang, Zhaoqian Liu

**Affiliations:** 1 Department of Clinical Pharmacology, Hunan Key Laboratory of Pharmacogenetics, and National Clinical Research Center for Geriatric Disorders, Xiangya Hospital, Central South University, Changsha 410008, Chinaying.gao@csu.edu.cn (Y.G.); wentingdai2023@csu.edu.cn (W.D.);; 2 Institute of Clinical Pharmacology, Engineering Research Center for Applied Technology of Pharmacogenomics of Ministry of Education, Central South University, Changsha 410078, China; 3 Department of Orthopaedics, Xiangya Hospital, Central South University, Changsha 410008, China; 4 Department of Pharmacy, Xiangya Hospital, Central South University, Changsha 410008, China; 5 Key Laboratory of Biological Nanotechnology of National Health Commission, Xiangya Hospital, Central South University, Changsha 410008, China

**Keywords:** PT-100, anti-PD-1, combination immunotherapy, tumor microenvironment, Lewis lung carcinoma

## Abstract

The remarkable efficacy of cancer immunotherapy has been established in several tumor types. Of the various immunotherapies, PD-1/PD-L1 inhibitors are most extensively used in the treatment of many cancers in clinics. These inhibitors restore the suppressed antitumor immune response and inhibit tumor progression by blocking the PD-1/PD-L1 signaling. However, the low response rate is a major limitation in the clinical application of PD-1/PD-L1 inhibitors. Therefore, combination strategies that enhance the response rate are the need of the hour. In this investigation, PT-100 (also referred to as Talabostat, Val-boroPro, and BXCL701), an orally administered and nonselective dipeptidyl peptidase inhibitor, not only augmented the effectiveness of anti-PD-1 therapy but also significantly improved T immune cell infiltration and reversed the immunosuppressive tumor microenvironment. The combination of PT-100 and anti-PD-1 antibody increased the number of CD4^+^ and CD8^+^ T cells. Moreover, the mRNA expression of T cell-associated molecules was elevated in the tumor microenvironment. The results further suggested that PT-100 dramatically reduced the ratio of tumor-associated macrophages. These findings provide a promising combination strategy for immunotherapy in lung cancer.

## 1. Introduction

Lung cancer is a common respiratory tumor. According to the latest Global Cancer Statistics 2020 lung cancer ranks second in terms of incidence and first in terms of mortality [1]. Lung cancer is categorized into non-small cell lung cancer (NSCLC) and small cell lung cancer, which account for 80% and 20% of the cases, respectively [2]. At present, the treatment options for lung cancer mainly include surgery, radiotherapy, chemotherapy, targeted therapy, and immunotherapy [3,4].

In recent years, owing to the development of oncology, immunology, molecular biology, and related disciplines, immunotherapy, represented by the use of immune checkpoint inhibitors, is gradually gaining prominence. Of the various immunotherapeutic approaches, PD-1/PD-L1 blocking therapy is the one that is most widely used. PD-1, a member of the CD28 superfamily of receptors, is expressed in various immune cells, including activated T cells, B cells, macrophages, regulatory T cells (Tregs), and natural killer (NK) cells. This receptor interacts with two ligands, namely, PD-L1 and PD-L2, which belong to the B7 family. The interaction of PD-1 with its ligands results in the inhibition of T cell activation and the consequent attenuation of T cell-mediated immune responses. Therefore, this inhibitor plays a crucial role in preventing the immune system from targeting normal cells. Malignant tumors exploit this regulatory pathway by overexpressing PD-L1/2 on their cell surfaces, which leads to diminished T-cell activation and immune response. Tumor cells are thus able to evade immune detection and destruction [5].

The immune escape of tumor cells via the upregulation of PD-L1 binding to PD-1 on T cells is a pertinent process in tumor progression. PD-1 inhibitors can block the combination of PD-1 and PD-L1/PD-L2, restore the attack and killing functions of inactivated T cells, and mobilize the body’s immune function to achieve antitumor effects. These inhibitors have demonstrated significant clinical effects and have become the new pillar of antitumor treatment [6]. Although an unprecedented, sustained response has been observed in some patients with the use of PD-1/PD-L1 inhibitor therapy, the low response rate in lung cancer remains an issue of concern. In most advanced solid tumors that are not limited to cancer types, the response rate of PD-1/PD-L1 inhibitors as a single agent is approximately 10–20%. Only <20% of the patients with NSCLC respond to immunotherapy alone, and a significant proportion of the patients develop resistance [7]. Therefore, identifying an effective combination therapy is crucial to improving the effectiveness of PD-1/PD-L1 inhibitors [8]. Researchers believe that combination therapy may be required for specific tumor types and that other drugs should be added to PD-1/PD-L1 inhibitors to improve the response rate, produce a lasting response, and thus achieve a better outcome [9].

PT-100, also known as Talabostat, Val-BoroPro, and BXCL701, is an oral nonselective dipeptidyl peptidase (DPP) inhibitor [10]. The targets of this drug include DPP4, DPP8, DPP9, and fibroblast activation protein (FAP). PT-100 has been shown to induce tumor regression in various preclinical tumor models. Adams et al. [11] observed that PT-100 did not exert any cytotoxic effects in vitro and that treatment of mice with this drug-induced tumor regression in various tumor models, such as fibrosarcoma, lymphoma, and melanoma. Furthermore, PT-100 upregulates mRNA expression of multiple cytokines with functions promoting innate and acquired immunity in tumor tissues and draining lymph nodes. Walsh et al. [12] reported that PT-100 caused tumor regression in bladder cancer models and that the possible mechanism is the acceleration of T cell priming. In addition, PT-100 has been clinically tested in healthy volunteers and patients with various tumors. Multiple doses of PT-100 were well-tolerated and exhibited a certain antitumor efficacy [13,14].

In this study, to determine if DPP inhibition can enhance the efficacy of PD-1/PD-L1 blockade, the therapeutic effect of an anti-PD-1 antibody combined with PT-100 was investigated in the Lewis lung cancer model. The findings indicated that combining PT-100 with anti-PD-1 antibody effectively inhibited tumor growth in the mouse model, improved the infiltration of immune cells in the tumor microenvironment (TME), and enhanced the mRNA expression of chemokines and cytokines. Furthermore, the combination regime altered the level of interferon (IFN)-γ in the serum of mice. Collectively, our data suggest that the combination regime displays a higher therapeutic efficacy than anti-PD-1 monotherapy in suppressing tumors. These findings are expected to provide a theoretical basis for the clinical use of the combination therapy of PD-1 inhibitor and PT-100 in treating lung cancer; however, more evidence from clinical trials is needed.

## 2. Materials and Methods

### 2.1. Chemicals and Reagents

The following compounds and reagents were used: PT-100 (HY-13233A) was purchased from MCE (Med Chem Express, South Brunswick Township, NJ, USA). Anti-mouse PD-1 mAb (CD279, BE0146) was obtained from Bio X cell (Lebanon, NH, USA). PT-100 was dissolved in saline and stored in a −20 °C freezer in aliquots. Anti-PD-1 mAb was dissolved in phosphate-buffered saline (PBS) and stored at 4 °C.

### 2.2. Animals

Eight-week-old female C57BL/6 mice were purchased from Hunan SJD Experimental Animal Company Limited (Changsha, China) and housed under specific pathogen-free conditions. The mice were given free access to food and water. Before the start of the experiment, the animals were acclimatized for approximately 1 week. All animal experiments were conducted in accordance with the guidelines of the Laboratory Animal Welfare Ethics Committee, Department of Laboratory Animals, Central South University (CSU), China.

### 2.3. Cell Lines and Tumor Models

A549, H1299, Lewis lung carcinoma (LLC), and RAW264.7 cells were obtained from the cell bank of the Chinese Academy of Sciences (Shanghai, China). The cells were cultured in Roswell Park Memorial Institute (RPMI)-1640 (Gibco, Thermo Fisher Scientific, Waltham, MA, USA) or Dulbecco’s modified eagle medium (Gibco, Thermo Fisher Scientific) supplemented with 10% fetal bovine serum and incubated at constant conditions of 37 °C and 5% CO_2_. To establish the subcutaneous tumor models, 5 × 10^5^ LLC cells in 100 mL of serum-free medium were injected subcutaneously into the right flank of the mice.

### 2.4. Cell Viability Assays

The effects of PT-100 on the viability of different cells were evaluated using the Cell Counting Kit-8 (CCK-8) assay. The cells were seeded in 96-well plates at an appropriate density and cultured at 37 °C for 24 h. Subsequently, the cells were treated with increasing concentrations of PT-100 for 72 h. After the addition of 10 μL of CCK-8 reagent to each well, the cells were incubated at 37 °C for 2–3 h. The absorbance of each well was determined at a wavelength of 450 nm using a microplate reader (BioTek, Winooski, VT, USA). The experiments were conducted in triplicate.

### 2.5. In Vivo Treatment

A total of 5 × 10^5^ LLC cells were injected subcutaneously into the right flank of C57/BL6J mice. When the average tumor volume reached approximately 50–100 mm^3^, tumor-bearing mice were randomly assigned to four treatment groups (*n* = 5/group): control, anti-PD-1, PT-100, and PT-100 + anti-PD-1. PT-100 (20 mg/kg) was administered by oral gavage daily, and anti-PD-1 antibody (200 μg) was intraperitoneally injected twice per week. Combination treatments with the PT-100 and anti-PD-1 antibody were administered under the same conditions as respective single-agent protocols. Administration began on day 9 after tumor cell injection and continued for 14 days until death. The mice were sacrificed on day 23. Tumor size was measured with a digital caliper every other day and used to calculate the tumor volume (mm^3^) [V = a × b^2^ × 0.52, a = the largest diameter, b = the smallest diameter]. The body weight was measured every other day, and the tumor weight was determined after the treatment by sacrificing the mice.

### 2.6. Flow Cytometry

Flow cytometry was performed using FACS CantoTM II (BD). Before staining, single-cell suspensions were prepared using the Tumor Dissociation Kit (Miltenyi Biotec, Tokyo, Japan). Single-cell suspensions were collected, and the cells were resuspended in PBS and the volume was adjusted to 100 μL. Subsequently, an appropriate amount of specific surface antibody was added to each sample and incubated for 20 min in the dark. The cells were then subjected to a cell membrane-breaking operation, and intracellular antibody staining was performed. Later, the samples were centrifuged at 300× *g* for 5 min, and the supernatant was discarded. Each sample was washed with 1 mL of PBS, resuspended in 300 μL of PBS, filtered, and assayed using the flow cytometer. All data were analyzed using the FlowJo 10.0 software. The following antibodies were used for flow cytometry: α-CD45 (30-F11, eBioscience, 0.2 mg/mL), α-CD3e (145-2C11, BD, 0.2 mg/mL), α-CD4 (GK1.5, BD, 0.2 mg/mL), α-CD8a (53-6.7, BD, 0.2 mg/mL), α-CD11b (M1/70, BD, 0.5 mg/mL), and α-F4/80 (T45-2342, BD, 0.2 mg/mL).

### 2.7. RNA Extraction and Real-Time PCR Analysis

Total RNA was extracted using the TRIzol reagent (Life Technologies, Carlsbad, CA, USA) according to the manufacturer’s protocol. RNA was reverse-transcribed into cDNA using the Prime Script RT Reagent Kit with gDNA Eraser (Takara Bio Inc., Kusatsu, Shiga, Japan). Real-time PCR was performed on a Light Cycler 96 System (Roche, Basel, Switerland) using TB green (Takara Bio Inc., Japan). Data were normalized to the housekeeping gene β-actin levels. The primers used in this study are listed in Table 1.

### 2.8. Immunohistochemical (IHC) Staining

The harvested tumor specimens were fixed, paraffin-embedded, and microtome-sectioned to 5-μm thickness. These sections were then incubated with primary antibodies targeting CD3 (clone ab16669, Abcam, Cambridge, UK) and CD34 (clone ab8158, Abcam, Cambridge, UK). Following the incubation with primary antibodies, the sections were rinsed thoroughly with PBS and subsequently incubated with mouse/rabbit secondary antibodies. The specimens were stained with 3,3′-diaminobenzidine and visualized microscopically. Images were captured and analyzed quantitatively using the ImageJ software (1.52 version). Positive immunoreactivity was identified based on the presence of distinct brown staining.

### 2.9. ELISA 

The level of IFN-γ in mouse serum was analyzed using the Mouse IFN-γ ELISA Kit (Elabscience, Wuhan, China) according to the manufacturer’s instructions.

### 2.10. Statistical Analysis

We determined the relative expression of selected genes using the 2^−ΔΔCt^ method. *Gapdh* was used as a reference gene. Statistical analyses were performed using the GraphPad Prism software (version 8.0.1; GraphPad Software), and all data were presented as mean ± standard error of the mean (SEM). The statistical significance of the differences between groups was analyzed using the unpaired t-test and one-way analysis of variance. A value of *p* < 0.05 was considered statistically significant.

## 3. Results

### 3.1. PT-100 Inhibited Macrophage Viability In Vitro

**Figure 1:**
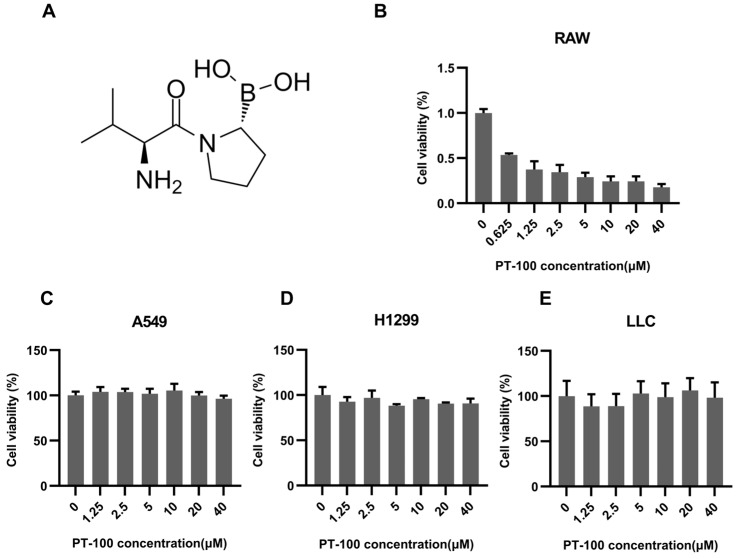
The cytotoxicity of PT-100 on different cells. (**A**) The structure of PT-100. (**B**–**E**) The cells were incubated with an increasing concentration of PT-100 for 72 h and the cell viability inhibition was analyzed by using a CCK-8 assay.

The structure of PT-100 is depicted in Figure 1A. The cytotoxic effects of PT-100 treatment on human lung cancer cells and RAW264.7 macrophages were initially tested. The results indicated that viability inhibition by PT-100 treatment occurred variably in these cell lines. PT-100 inhibited the viability of RAW264.7 cells in a dose-dependent manner (Figure 1B), but in other lung cancer cell lines, no significant inhibition was observed (Figure 1C–E).

### 3.2. Combination Therapy of PT-100 with Anti-PD-1 Antibody Potently Inhibited Tumor Growth

To explore whether the combination of PT-100 with anti-PD-1 antibody exerted a better antitumor effect than monotherapy, a xenograft model of LLC was developed in C57BL/6J mice. The tumor-bearing mice were randomly categorized into four groups: control group, anti-PD-1 group, PT-100 group, and PT-100 + anti-PD-1 group. 

**Figure 2:**
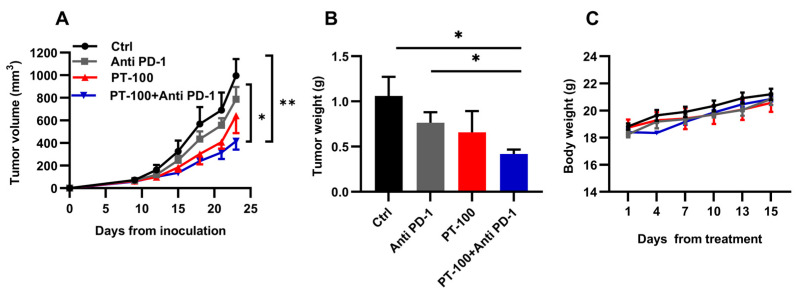
The antitumor efficacy of PT-100 and PD-1 blockade in mice. Mice-bearing LLC tumors received vehicle, anti-PD-1 antibody (100 μg), PT-100 (20 mg/kg·d), or a combination of these. (**A**) Tumor growth curve. (**B**) After the mice were sacrificed, their tumor tissues were separated and weighed. (**C**) Body weights of mice during the treatments. * *p* < 0.05, ** *p* < 0.01.

Compared with the control group, tumor growth was attenuated in mice that received anti-PD-1 antibody or PT-100 monotherapy. When PT-100 and anti-PD-1 antibody were combined, the inhibitory effect was significantly enhanced (Figure 2A), resulting in a decrease in tumor weight (Figure 2B). In terms of body weight, no significant decrease was noted in any of the groups during the administration. All groups exhibited a steady increase in body weight (Figure 2C), which suggests that the dosing regimens did not cause major side effects.

### 3.3. Combination of PT-100 with Anti-PD-1 Antibody Increased the Infiltration of CD4^+^ and CD8^+^ T Cells in the TME 

**Figure 3:**
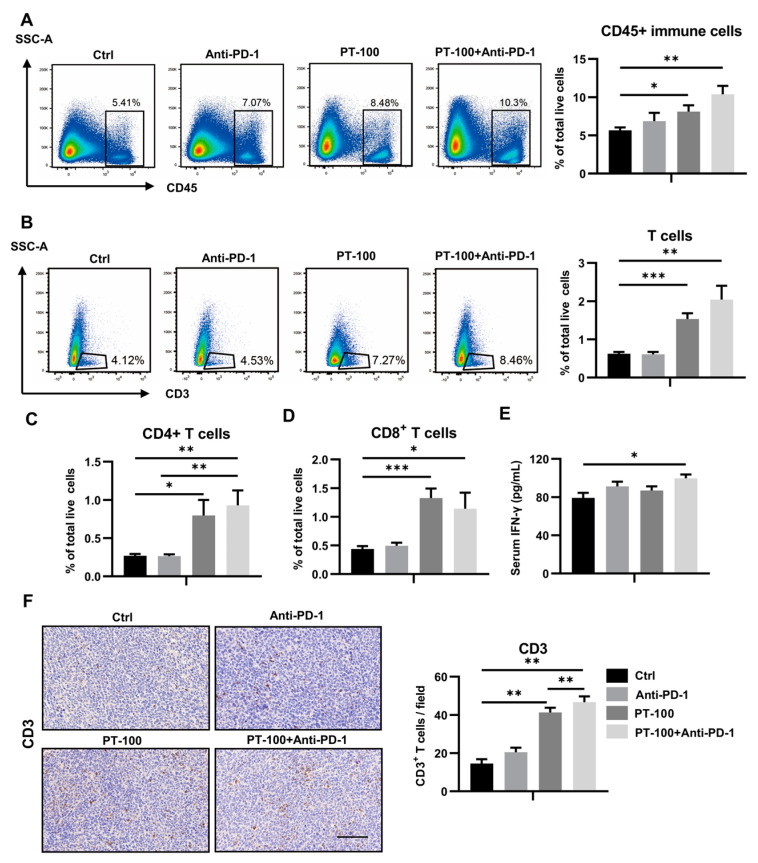
PT-100 combination with PD-1 blockade treatment increases the infiltration of T cells in vivo. The day after the last injection, the mice were sacrficed and their tumors were isolated. The isolated tumors were then digested to prepare single-cell suspensions and the tumor-infiltrating immune cells were analyzed by flow cytometry. (**A**,**B**) Flow-cytometry analysis of CD45^+^ immune cells and CD3^+^ T cells. (**C**,**D**) Flow cytometry analysis of the proportion of T-cell subsets CD4^+^ T cells and CD8^+^ T cells. (**E**) The serum IFN-γ level in different treatment groups after therapy. Before sacrificing the tumor-bearing mice, blood was extracted and the concentration of serum cytokines was examined by ELISA. The unit is pg/mL. (**F**) The tumor sections were stained for T-cell marker CD3 by IHC. The positive cells were counted. From each slide, 8 fields were selected for analysis. Scale bar = 100 μm. * *p* < 0.05, ** *p* < 0.01, *** *p* < 0.001.

Previous studies have established that the therapeutic effect of PD-1/PD-L1 inhibitors is largely dependent on the extent and magnitude of T cell response within the TME [15], including CD4^+^ T cells and CD8^+^ T cells. Flow cytometry was used to determine the population of T cells and their subsets in each administration group. Initially, anti-PD-1 and PT-100 treatments were found to slightly increase the total number of immune cells (CD45^+^), whereas anti-PD-1 antibody combined with PT-100 caused a more significant enhancement of CD45^+^ immune cells (Figure 3A). Furthermore, T-cell infiltration in each group was investigated, which revealed that it was not significantly increased in the PD-1 group. However, it was substantially elevated after PT-100 administration and was further increased in the combination group (Figure 3B). IHC staining demonstrated a similar trend (Figure 3F). In addition, the infiltration of CD4^+^ T cells and CD8^+^ T cells was significantly enhanced in the combination group (Figure 3C,D). Next, ELISA was used to examine the expression levels of serum IFN-γ to better understand how the immune system responds to the treatments. As shown in Figure 3E, the serum level of IFN-γ in the combination group was significantly higher than that in the control group.

### 3.4. Combination of PT-100 with Anti-PD-1 Antibody Reduced the Infiltration of Tumor-Associated Macrophages (TAMs)

**Figure 4:**
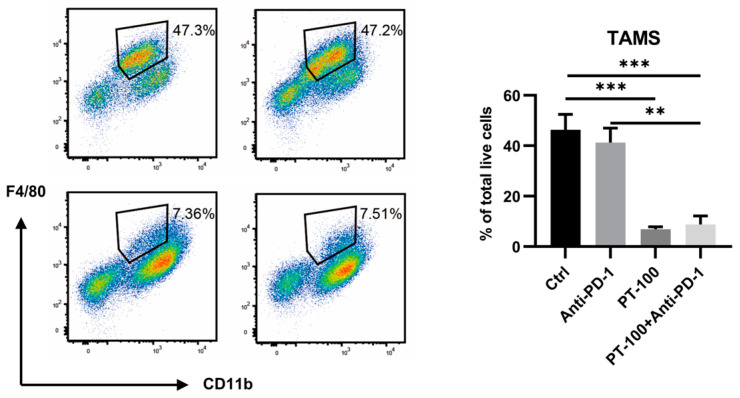
The effects of anti-PD-1 and PT-100 on TAM proportions. PT-100 or combination treatment substantially decreased the infiltration of TAMs. The percentage of TAMs in LLC tumor, gated on CD45+ cells. ** *p* < 0.01, *** *p* < 0.001.

There is substantial evidence that TAMs can promote the malignant phenotype of tumors. TAMs play a pertinent role in enhancing tumor progression and metastasis and also exhibit immunosuppressive activity. Flow cytometry was used to examine the proportion of TAMs in each treatment group [16,17]. Compared with the control group, no significant changes in the proportions of TAMs were noted in the PD-1 alone group. However, the proportions of TAMs were considerably reduced in the PT-100 group and the combination group (Figure 4). This finding indicates that PT-100 has a strong scavenging effect on TAMs and immensely reduces the total number of TAMs in the tumor tissue.

### 3.5. Characterization of Chemokines and Cytokines in Tumors Treated with Combination Therapy 

**Figure 5:**
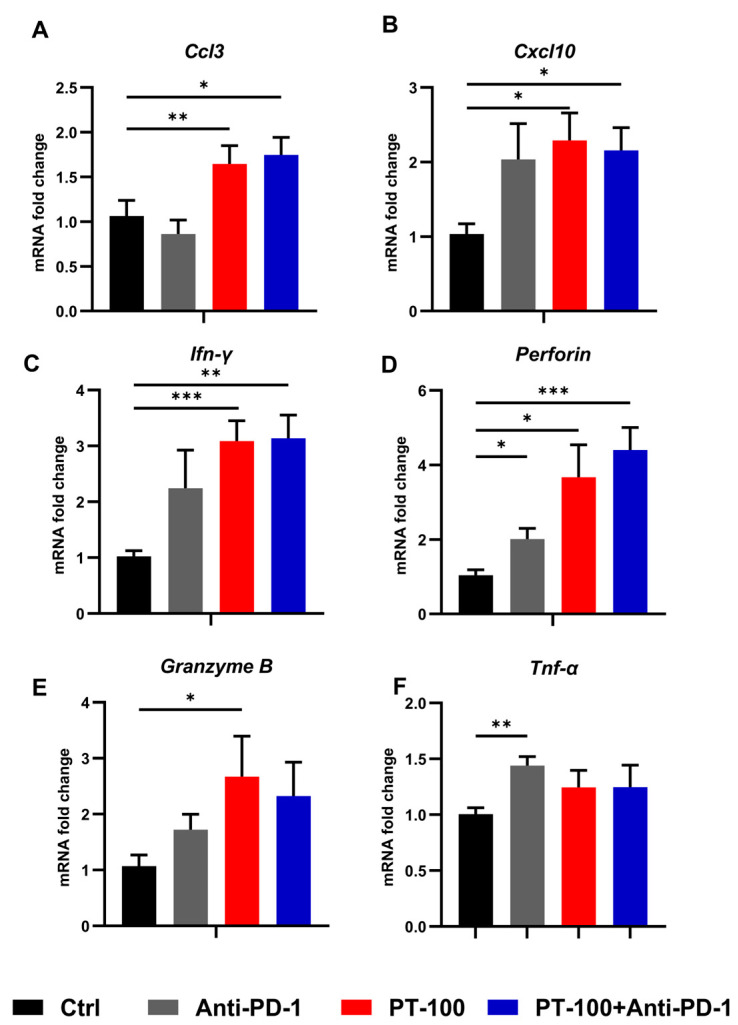
T -cell recruitment-related and T -cell response-related genes were upregulated in tumors that received PT-100 and/or PD-1 blockade treatment. After the mice were sacrificed, their tumors were isolated and RNA was extracted to detect mRNA expression. T -cell recruitment-related genes: (**A**) Ccl3, (**B**) Cxcl10. T -cell response-related genes: (**C**) Ifn-γ, (**D**) Perforin, (**E**) Tnf-α, and (**F**) Granzyme B by qPCR. * *p* < 0.05, ** *p* < 0.01, *** *p* < 0.001.

To further explore the detailed mechanism of the antitumor effects of T cells, the mRNA expression levels of genes associated with T cell recruitment (*Ccl3* and *Cxcl10*) and T cell activation or response (*Ifn-γ*, *Perforin*, *Tnf-α*, and *Granzyme B*) were investigated. As shown in Figure 5A,B, the expressions of T cell-recruiting chemokine genes *Ccl3* and *Cxcl10* were significantly upregulated in the monotherapy and combination therapy groups. Regarding changes in the mRNA expression of T cell-recruiting chemokine genes, a considerable increase was noted in *Ccl3* expression in mouse tumor tissues after PT-100 or combination therapy, whereas *Cxcl10* expression was substantially elevated in the PT-100, anti-PD-1 group, and combination groups. Moreover, the cytokine gene expression of IFN-γ, perforin, TNF-α, and granzyme B were upregulated to varying degrees in each administration group. Specifically, the PT-100 group exhibited a more pronounced effect on IFN-γ and granzyme B expression (Figure 5C–F).

### 3.6. Influence of Combination Therapy on Angiogenesis

**Figure 6:**
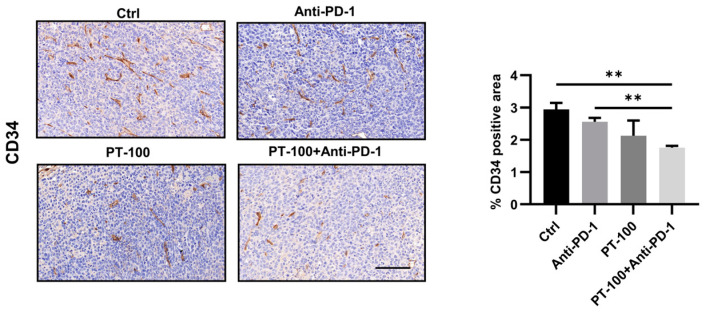
The effects of anti-PD-1 and PT-100 on tumor angiogenesis. The expression of the tumor vascular endothelial cells marker, CD34, was identified by IHC in the harvested tumors. Tumor angiogenesis is represented by the ratio of CD34-positive area. From each slide, 8 fields were selected for analysis and the data were quantitated to represent the mean ± SEM. Scale bar = 100 μm. ** *p* < 0.01.

Abnormal angiogenesis is one of the major features of the TME [18]. Angiogenesis promotes tumor growth and metastasis and plays a key role in the development of an immunosuppressive microenvironment, leading to immunotherapeutic resistance. The CD34 expression was determined using IHC staining to quantify the angiogenesis of tumors. CD34 is a marker of vascular endothelial cells, and angiogenesis is represented by the proportion of the positive area. The findings revealed that tumor angiogenesis was significantly inhibited in the combination group (Figure 6).

## 4. Discussion

PD-1/PD-L1 blockade therapy is presently the most widely used clinical immunotherapy [19,20]. Although treatment with PD-1/PD-L1 inhibitors significantly prolongs the overall survival of the patient, the low response rate and the prevalence of drug resistance limit their clinical use in NSCLC. Combining PD-1/PD-L1 inhibitors with other antitumor therapies may be a viable strategy to augment the efficacy of these inhibitors and elicit durable immune responses. This study observed that DPP inhibition using PT-100 reduced tumor growth and increased anti-PD-1 efficacy in the LLC model. Mechanistically, combination therapy can regulate T -cell infiltration in the TME and enhance the expressions of chemokines and cytokines. The immunosuppressive state of the TME is thus improved. In this study, the combined treatment significantly reduced the number of TAMs that promote angiogenesis and inhibit antitumor immunity. In addition, tumor angiogenesis and serum IFN-γ levels were altered by the combination regime.

One of the factors that causes resistance to PD-1/PD-L1 therapy is the presence of an immunosuppressive microenvironment in the tumor. PD-1/PD-L1 therapy aims to restore suppressed T cell activation and allow activated T cells to kill tumor cells. However, several immunosuppressive components occur in the TME, which can inhibit tumor killing by T cells. Therefore, transforming the immunosuppressive microenvironment into an immune-friendly microenvironment may enhance the response of PD-1/PD-L1 inhibitors. The efficacy of immunotherapy is known to depend on the tumor-infiltrating lymphocytes (TILs) in the TME [21]. In this study, the TILs in each group were studied, which indicated an increase in the infiltration of CD45^+^ immune cells, CD4^+^ T cells, and CD8^+^ T cells in the combination group. Furthermore, the combination therapy enriched the genes associated with T cell recruitment and T cell activation or response. These findings signify that the immunosuppressive microenvironment has been partly reversed. These effects could be attributed to the targets inhibited by PT-100, namely, DPP4 and DPP8/9.

A previous study examining the impact of specific DPP4 inhibition in murine cancer models has demonstrated that DPP4 inhibition not only elevates tumor CXCL10 levels but also enhances the trafficking of CXCR3^+^ NK cells and CD8^+^ T cells into tumors. DPP4 can drastically influence the activity of chemokines by truncating them at certain truncation sites. Consequently, tumor immunity may change significantly owing to DPP4 inhibition [22]. Da Silva et al. [23] confirmed that the inhibition of DPP4 enzymatic activity can preserve biologically active CXCL10 and increase lymphocyte trafficking into the tumor in vivo. This finding agrees with our observation that inhibition using PT-100 augmented the expression level of CXCL10 and the number of T cells.

High levels of TAMs in human tumors are often correlated with reduced patient survival. Studies in mice have shown that TAMs promote tumor progression and suppression of adaptive antitumor immunity. Moreover, TAMs modify or limit the effectiveness of certain anticancer therapies [24]. It has been well established that PT-100 can trigger pyroptosis of monocytes and macrophages by inhibiting DPP8/DPP9 [25]. Pyroptosis is a newly discovered proinflammatory form of programmed cell death [26] and is mediated by the gasdermin family. Pyroptosis is accompanied by cell swelling and lysis, causing the release of cytokines, stimulating innate immunity and adaptive immunity, and transforming “cold” tumors into “hot” tumors that are suitable for immunotherapy. Under certain circumstances, exogenous activation of pyroptosis can induce potent antitumor immunity and also act synergistically with immunotherapy [27]. The experiments further demonstrated that PT-100 causes macrophage death in vivo and that the viability of macrophages continued to decline as the concentration of PT-100 increased in vitro. This decrease was hypothesized to be related to the inhibition of DPP8/9-induced macrophage pyroptosis. Okondo et al. [25] identified that PT-100 was the first small molecule shown to have the ability to induce pyroptosis based on the principle that the inhibition of two serine proteases, DPP8 and DPP9, by PT-100, activates the procaspase-1 form of caspase-1, independent of the inflammasome junction ASC. Activated procaspase-1 does not efficiently process itself or IL-1β but cleaves and activates gasdermin D to trigger pyroptosis. PT-100 facilitates the elimination of TAMs from the TME through a mechanism that triggers pyroptosis and the production of IL-1β and IL-18. This process culminates in the release of cytokines, effectively stimulating both innate and adaptive immunity. Consequently, this action modestly mitigates the immunosuppressive nature of the TME.

In tumors, angiogenesis is perpetuated by the overexpression of pro-angiogenic factors, resulting in the formation of blood vessels that are tortuous, entangled, abnormally enlarged, and disorganized. Abnormal tumor angiogenesis creates hypoperfusion, hypoxia, and other circumstances that impede immune cell function and eventually lead to an immunosuppressive microenvironment. Antiangiogenic therapy may therefore be effective in enhancing immunotherapy [28]. Of the various targets of PT-100, FAP exhibits enzymatic activity and can assist tumor cell infiltration and invasion by participating in the remodeling of intercellular substances [29]. Furthermore, overexpression of FAP has been reported to promote tumor microvascular formation [30], which suggests that it may be able to stimulate tumor angiogenesis. Hence, PT-100 was hypothesized to reduce intratumor angiogenesis by inhibiting FAP. Analysis of the staining results of the vascular marker CD31 in tumor tissues confirmed that PT-100 administration reduced angiogenesis in tumors. This phenomenon may also serve as a significant rationale behind the enhancement of the immune microenvironment by PT-100.

Previous studies have confirmed that PT-100 induces tumor regression in numerous models, such as fibrosarcoma, lymphoma, and melanoma. Moreover, clinical trials of PT-100 in combination with certain chemotherapeutic drugs have been conducted, but these demonstrated limited improvement compared with monotherapy [31,32]. Today, this field has regained the interest of researchers. Recently, the combination of BXCL701 and immune checkpoint inhibitors has been reported to exert synergistic antitumor effects in syngeneic pancreatic ductal adenocarcinoma models [33,34]. In a mouse model of pancreatic cancer, the triple therapy of BXCL701, NKTR-214, and PD-1 inhibitor achieved tumor regression in all mice [35]. Based on these findings, we propose that combining BXCL701 with immunotherapy may boost its clinical efficacy in lung cancer. Nonetheless, further clinical studies are required to validate the efficacy of the combination therapy.

## 5. Conclusions

This study revealed that DPP inhibition using PT-100 can cause the death of macrophages and improve anti-PD-1 efficacy in an LCC model, which could be attributed to improvements in the immunosuppressive microenvironment. This improvement was achieved by enhancing the proportion of T cells, decreasing the proportion of TAMs, and reducing angiogenesis. In clinical scenarios, the combined application of PT-100 and PD-1 inhibitors may be an effective antitumor therapy for lung cancer. However, more clinical evidence is required to determine the percentage of patients who would benefit from this regime.

## Figures and Tables

**Table 1:** Primers used in this study.

Gene	Primer Pair	Primer Sequence (5′-3′)
*Ccl* *3*	forward	TTCTCTGTACCATGACACTCTGC
	reverse	CGTGGAATCTTCCGGCTGTAG
*C* *xcl* *10*	forward	CCAAGTGCTGCCGTCATTTTC
	reverse	GGCTCGCAGGGATGATTTCAA
*T* *nf* *-α*	forward	CAGGCGGTGCCTATGTCTC
	reverse	CGATCACCCCGAAGTTCAGTAG
*I* *fn* *-γ*	forward	ATGAACGCTACACACTGCATC
	reverse	CCATCCTTTTGCCAGTTCCTC
*Granzyme B*	forward	TCATGCTGCTAAAGCTGAAGAG
	reverse	CCCGCACATATCTGATTGGTTT
*Perforin*	forward	CAAGGTAGCCAATTTTGCAGC
	reverse	GTACATGCGACACTCTACTGTG

## Data Availability

Datasets of the current study are not publicly available but are available from the corresponding author on reasonable request.
